# A Not-So-Sweet Crohn's Disease: A Case Report of Ileocecal Crohn's Disease Unmasked by Sweet Syndrome

**DOI:** 10.1155/carm/6680526

**Published:** 2025-04-21

**Authors:** Amir Omar, Rayane Salameh, Karam Karam, Chakib Khoury, Elias Fiani

**Affiliations:** ^1^Department of Gastroenterology, University of Balamand, Beirut, Lebanon; ^2^Department of Internal Medicine, University of Balamand, Beirut, Lebanon

**Keywords:** acute febrile neutrophilic dermatosis, Crohn's disease, inflammatory bowel disease, Sweet syndrome, systemic corticosteroids

## Abstract

Sweet syndrome (SS) is an acute febrile neutrophilic dermatosis characterized by a constellation of clinical symptoms and histologic findings: pyrexia, neutrophilia, and tender and erythematous cutaneous lesions (plaques, nodules, and papules) with neutrophilic infiltration of the upper reticular dermis. SS can be either an inflammatory disease or a hypersensitivity disorder. SS has been associated with autoimmune disease, such as inflammatory bowel disease (IBD), rheumatoid arthritis (RA), systemic lupus erythematosus (SLE), and sarcoidosis. We herein present a case of a 70-year-old white male presenting for persistent fevers, rash, intermittent diarrhea, and hematochezia. The patient had marked improvement of his clinical symptoms following systemic corticosteroid treatment. He was diagnosed with SS and ileocecal Crohn's disease (CD). This article highlights the need to rule out CD in the setting of SS and gastrointestinal (GI) manifestations.

**Summary**

• Learning points
• The patient in this case met all criteria for the diagnosis of classical SS, which heralded an undiagnosed ileocecal CD.
• Thus, it has become clear that SS does not always parallel CD; rather, SS can unmask an underlying CD.

## 1. Introduction

Crohn's disease (CD) is an inflammatory condition that can affect any portion of the gastrointestinal (GI) tract. Patients with inflammatory bowel disease (IBD) may experience persistent inflammation in organs other than the GI system. Extraintestinal manifestations (EIMs) are most commonly found in the eyes, skin, and joints and have been documented to affect 6%–47% of people with CD and ulcerative colitis (UC) [1].

Mucocutaneous symptoms are seen in 22% and 75% of people with CD and between 5% and 11% of patients with UC [2]. The most prevalent mucocutaneous manifestations associated with IBD are erythema nodosum (EN) and pyoderma gangrenosum (PG) [1].

Sweet syndrome (SS), an eponym for acute febrile neutrophilic dermatosis, is a rare cutaneous manifestation of IBD with paucity of data on its prevalence and pathogenesis [1, 3–5].

SS is a challenging diagnosis as it has a constellation of clinical symptoms, histopathologic findings, and physical features.

Histologic findings are characterized by dense neutrophilic infiltrates in the upper dermis.

SS is categorized into three subtypes: classic SS (CSS), malignancy-associated SS (MASS), and drug-induced SS (DISS).

CSS manifests as pyrexia, increased neutrophilic count, tender and erythematous skin lesions, and diffuse infiltration of neutrophils in the upper dermis. CSS has 2 major criteria and 4 minor criteria (Table 1). Both major criteria and at 2 out of 4 minor criteria should be met for the diagnosis of CSS.

The pathogenesis of SS is multifactorial, and systemic corticosteroids remain the gold standard for the treatment of the disease. Patients with SS respond promptly to corticosteroids with normalization of inflammatory markers and subsidence of fevers and cutaneous lesions. Skin lesions subside without scarring. CSS can be a cutaneous harbinger for an undiagnosed CD. We describe a case of CSS that uncovered a diagnosis of ileocecal CD.

## 2. Case Presentation

A 70-year-old white man, previously healthy, presented to the emergency department for an abrupt onset of high-grade fevers, night sweats, and rash of 1-week duration. The rash was covering his back, shoulders, and torso. The patient described the rash as “burning-like” but nonpruritic.

Review of systems was pertinent for hematochezia and an intermittent watery diarrhea of 4-5 times daily during the last month along with mild diffuse abdominal discomfort. The patient was not on any medications known to be associated with DISS. He denies any arthralgia, myalgias, or headache. He also denies any previous GI infection or upper respiratory tract infection. He had no recent history of vaccination. He has no history of hematologic or visceral malignancies. He is a nonsmoker and does not consume alcohol.

The patient was febrile with a body temperature of 39.5°C with a heart rate of 115 beats per minute. His blood pressure was normal.

**Figure 1:**
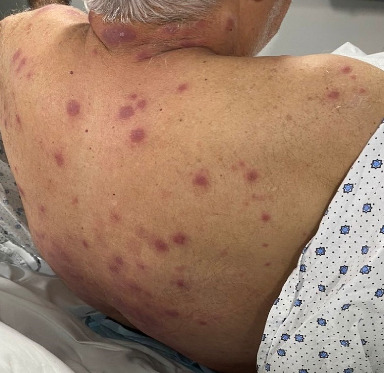
Erythematous and tender maculopapular cutaneous lesions.

Upon physical examination, the skin revealed tender, edematous, and brightly erythematous plaques over his scalp, neck, trunk, and back that did not follow a particular dermatome (Figure 1).

The patient exhibited diffuse abdominal tenderness upon palpation that was more localized to the right upper quadrant. The remainder of the physical exam was unremarkable.

Laboratory tests revealed a high white blood cell (WBC) count of 20,000/mm^3^ with neutrophilia. His C-reactive protein (CRP) was 17 mg/dL (normal values between 0.3 and 1.0 mg/dL), and his erythrocyte sedimentation rate (ESR) was 100 mm/hr (normal value less than 20 mm/hr). Renal and liver function tests were normal.

Tests for antineutrophil cytoplasmic antibody (ANCA), rheumatoid factor (RF), anti-cyclic citrullinated peptide (CCP), anti-double-stranded DNA (ds-DNA), and antinuclear antibody (ANA) were negative. Infectious workups including bacterial (aerobic and anaerobic), viral, fungal, and mycobacterial cultures were negative. QuantiFERON-TB Gold, parvovirus, *Bartonella*, and *Brucella* antibody titers were negative. The remainder of the rheumatologic workup and complement levels were normal.

Transthoracic echocardiography (TTE) and transesophageal echocardiography (TEE) demonstrated normal cardiac valves with no vegetations.

Computed tomography (CT) scan of the abdomen and pelvis with intravenous (IV) contrast showed mild distention with wall thickening and edema of the distal small bowel loops, mainly at the level of the terminal ileum, suggesting an infectious ileitis vs. IBD, with no evidence of small bowel obstruction or stenosis.

The patient was initially started on broad-spectrum IV antibiotics (vancomycin and piperacillin/tazobactam) without resolution of fevers and in the setting of a negative infectious workup. Therefore, IV antibiotics were discontinued.

A 4 mm lesional skin biopsy of a macular nodule on his back revealed a neutrophilic infiltrate in the dermis and subcutaneous tissues without evidence of leukocytoclastic vasculitis. Thus, a diagnosis of SS was made.

Thereafter, the patient was started on IV methylprednisolone at a dosage of 50 mg for 3 days with dramatic improvement of fevers and skin lesions. He had a marked decrease in CRP and resolution of leukocytosis. The patient was then switched to oral prednisone at a dose of 1 mg/kg daily.

**Figure 2:**
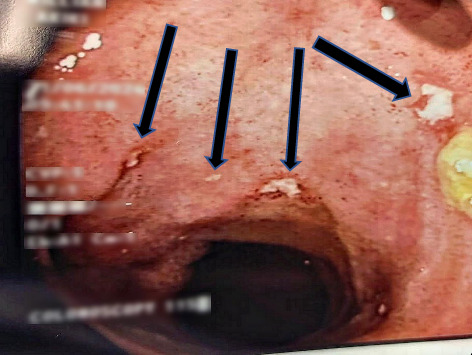
An ileocolonoscopy revealing superficial aphthous ulcerations (black arrows) interspersed with normal mucosa at the level of the terminal ileum.

Owing to persistent hematochezia and diarrhea along with a high fecal calprotectin level (1000 Ug/g), a colonoscopy was opted for to exclude an underlying IBD etiology. A colonoscopy with intubation of the terminal ileum was subsequently performed showing superficial aphthous ulcerations interspersed with normal mucosa along with erythematous cecal mucosa (Figure 2). Of note, water-assisted colonoscopy (WAC) was performed using water immersion and exchange technique for enhanced endoscopic visualization and decreased postprocedural pain. Biopsies taken from the terminal ileum and cecum revealed chronic active ileitis and colitis with signs of cryptitis and noncaseating granulomas, favoring a diagnosis of CSS in the setting of Crohn's ileocolitis.

The patient was discharged home with a prolonged course of prednisone taper. He was started on adalimumab after 2 months of completing his prednisone taper with an induction dose of 160 mg followed by 80 mg, then 40 mg every 2 weeks. His GI symptoms continued to improve on scheduled adalimumab therapy with a Crohn's Disease Activity Index (CDAI) of less than 150.

## 3. Discussion

SS was initially identified by Dr. Robert Douglas Sweet in 1964 [4–6]. SS is an acute febrile neutrophilic dermatosis that can be either an inflammatory disease or a hypersensitivity reaction. SS can be autoimmune in nature and often presents with a constellation of clinical symptoms and histologic findings. Skin biopsy reveals abundant neutrophils in the upper reticular dermis with endothelial cells edema in the absence of leukocytoclastic vasculitis.

Clinically, SS is characterized by the abrupt onset of tender and erythematous lesions, pyrexia, and neutrophilic leukocytosis. The cutaneous lesions are often nonpruritic, tender, and painful. They can be erythematous, waxy, and violaceous. Papules and nodules can coalesce to form larger plaques [4, 5, 7]. SS has been classified into three clinical subtypes: CSS, MASS, and DISS [7–9].

CSS is often preceded by a GI or an upper respiratory tract infection. CSS has been associated with IBD, such as CD and UC. SS is more commonly seen in patients with CD (70%) as opposed to patients with UC (30%) [4, 5, 7, 10]. The etiopathogenesis of SS remains unclear, and it is considered to be multifactorial. It was presumed that certain drugs and diseases can elicit a hypersensitivity reaction mediated by cytokines, resulting in neutrophil activation with subsequent fever and neutrophilic infiltration of the dermis [7, 10]. There has been a link between the melanocortin system and IBD underscoring a potential association between MC3R and MC5R expression and IBD activity, with higher receptor expression being associated with a more severe disease activity [11].

The diagnosis of SS is made when two major criteria and at least 2 minor criteria are met (Table 1) [5, 10].

Our patient met all major and minor criteria. The patient did not improve following treatment with broad-spectrum antibiotics. An infectious etiology was excluded as the extensive infectious workup and cultures were negative. Our patient exhibited a dramatic response to corticosteroids with complete resolution of skin lesions and fevers and resultant normalization of inflammatory markers.

The relationship between SS and IBD activity is not well-defined; however, it parallels IBD activity in 67%–80% of cases [5, 7].

Since most of the cutaneous manifestations of IBD parallel disease activity, it is reasonable that patients who were diagnosed with IBD after the onset of SS had mildly active or asymptomatic IBD at that time [5]. Multiple studies demonstrated that SS is a heralding sign of IBD, while on the other hand, other studies showed that it is an EIM occurring later after the diagnosis of IBD [1, 9, 10]. Skin lesions and symptoms of SS respond favorably to systemic corticosteroids (prednisone 1 mg/kg/day or equivalent, tapered to 10 mg/day within 4–6 weeks). Other first-line treatments include colchicine (0.5 mg orally three times a day for 10–21 days) and potassium iodide (300 mg orally three times a day) [3, 5].

The second-line therapy consists of indomethacin or immunosuppressants, such as cyclosporine. Antitumor necrosis factor and cyclosporine have shown success in the management of refractory SS cases [3, 5].

In summary, this article describes a case of CSS as an initial presentation of ileocecal CD. The patient had hematochezia and diarrhea with ileocolonoscopy and biopsies confirming a diagnosis of CD. It is established in the medical literature that CSS parallels the activity of CD; however, in this article, CD surfaced cutaneously as CSS. Thus, a prompt diagnosis of CD should be made in the setting of CSS and GI manifestations. The patient had remarkable responsiveness to corticosteroid treatment and improved GI symptoms following scheduled adalimumab therapy. Thus, a prompt diagnosis of CSS-induced CD is crucial for timely management and better clinical, endoscopic, and histologic outcomes.

## 4. Conclusion

The diagnosis of SS remains a daunting challenge as it is a diagnosis of exclusion. A negative infectious workup points to an autoimmune etiology. Lesional skin biopsies and a high clinical index of suspicion are conducive to the diagnosis of SS. Systemic corticosteroids are the therapeutic mainstay for SS. The remarkable improvement of the patient with steroids in terms of clinical symptoms and laboratory markers confirms the diagnosis of CSS. Our patient met all criteria for the diagnosis of CSS, which heralded an undiagnosed ileocecal CD. Thus, it has become clear that SS does not always parallel CD; rather, SS can unmask an underlying CD.

## Figures and Tables

**Table 1:** Diagnostic criteria for classical Sweet syndrome (CSS): both major criteria and two of the four minor criteria should be met for the diagnosis of CSS.

Major criteria	Minor criteria
Abrupt skin eruption of erythematous and tender lesions (macule, papule, plaque)	Fever/pyrexia
Dense neutrophilic infiltrates in the upper reticular dermis in the absence of leukocytoclastic vasculitis	Association with IBD, pregnancy, hematologic or visceral malignancy, or previous vaccination or preceded by GI infection or upper respiratory infection
	Marked responsiveness to systemic corticosteroids
	3 of 4 abnormal laboratory values at presentation: elevated CRP, ESR > 20 mm/hr, WBC > 8000, and neutrophils > 70%

Abbreviations: CRP = C-reactive protein; ESR = erythrocyte sedimentation rate; GI = gastrointestinal; IBD = inflammatory bowel disease; WBC = white blood cell.

## Data Availability

The data that support the findings of this study are available from the corresponding author upon reasonable request.
